# The adjuvant therapy of edible herbal product including colchicum bulb, olive leaf, black cumin seeds, lavender flower, and ginger rhizome on the outcome of patients with severe and critical COVID-19: A double-blind randomized controlled clinical trial

**DOI:** 10.22038/AJP.2024.24633

**Published:** 2024

**Authors:** Vahid Sebghatollahi, Mansour Siavash Dastjerdi, Farzin Ghiasi, Babak Alikiaii, Morteza Pourahmad, Afsaneh Yegdaneh, Mojtaba Akbari, Reza Ghadiri

**Affiliations:** 1 *Department of Gastroenterology and Hepatology, Isfahan University of Medical Sciences, Isfahan, Iran*; 2 *Department of Endocrine and Metabolism, Isfahan Endocrine and Metabolism Research Center, Isfahan University of Medical Sciences, Isfahan, Iran*; 3 *Department of Pulmonary Diseases, Isfahan University of Medical Sciences, Isfahan, Iran*; 4 *Department of Anesthesiology, Anesthesiology and Critical Care Research Center, Isfahan University of Medical Sciences, Isfahan, Iran*; 5 *Department of Infectious Disease and Tropical Medicine, Nosocomial Infection Research Center, Isfahan University of Medical Sciences, Isfahan, Iran*; 6 *Department of Pharmacognosy, School of Pharmacy and Pharmaceutical Sciences, Pharmaceutical Sciences Research Center, Isfahan University of Medical Sciences, Isfahan, Iran*; 7 *Department of Epidemiology, Isfahan Endocrine and Metabolism Research Center, Isfahan University of Medical Sciences, Isfahan, Iran*; 8 *Department of Traditional Medicine, Isfahan University of Medical Sciences, Isfahan, Iran*

**Keywords:** COVID-19, Colchicum autumnal L., Olive, Nigella sativa L., Lavandula angustifolia L., Zingiber

## Abstract

**Objective:**

The present study aimed at evaluating the effect of combination of medicinal plants, including *Colchicum autumnal *L., *Olea europaea *L.*,* *Nigella sativa *L., *Lavandula angustifolia* L., and *Zingiber officinale *Roscoe, on the recovery and outcome of COVID-19 patients.

**Materials and Methods:**

This study was conducted on 150 COVID-19 patients. All patients received both pharmaceutical and supportive treatments. In addition to the standard care treatment, intervention group received two capsules of herbal medicine orally every 8 hours, while control group received placebo.

**Results:**

Oxygen saturation percentage (SpO2) of the intervention group (median:88.00) was significantly higher than that of the control group (median:86.00), while C-reactive protein (CRP) of the intervention group (median:20.00) was significantly lower than that of the control group (median:28.00) at the time of hospital discharge (p<0.05).

**Conclusion:**

The combination of studied medicinal plants could significantly reduce the oxygen requirement and oxygen therapy.

## Introduction

Severe acute respiratory syndrome coronavirus 2 (SARS-CoV-2) was identified in China in late December 2019, then introduced as the causative agent of the COVID-19 disease, and declared as a global pandemic. Most people infected with this virus have symptoms such as fever, chills, body aches, weakness, and lethargy and sometimes experience respiratory or digestive symptoms. A number of patients have severe respiratory or systemic symptoms that can lead to death. SARS-CoV-2 virus belonging to the coronavirus family enters the cell through the respiratory system, which hosts Angiotensin-converting enzyme (ACE) receptors on the respiratory epithelium (Ahmadi et al., 2021). Bronchi, bronchial glands, monocytes, and alveolar macrophages are present in the alveolar epithelial cells (Lockyer et al., 2015). Epithelial cells release pro-inflammatory cytokines such as interleukin (IL)-1, IL-6, and tumor necrosis factor (TNF) when faced with the virus. This release causes neutrophils to migrate towards the pathogens, bind to the epithelial cells, and secrete various cytokines such that a cytokine storm occurs in severe cases of the disease (Koshak and Koshak, 2020). Cytokines increase the vascular permeability, and facilitate the release of immune cells. The mentioned course causes the inflammation process to progress at a high speed and intensity (Hu et al., 2021).

Considering the high prevalence of this disease, its progressive speed, and its high mortality rate, numerous studies have been conducted in different countries regarding the prevention and treatment of this emerging disease. At present, COVID-19 treatment is based on the use of a) anti-inflammatory drugs including corticosteroids (dexamethasone and prednisone), colchicine, hydroxychloroquine, and chloroquine, b) non-steroidal anti-inflammatory drugs (NSAIDs) such as naproxen, c) anticoagulants such as heparin and enoxaparin, d) antiviral drugs such as remdesivir, e) supportive treatment, and f) oxygen therapy (Shahidi et al., 2022; Ahmadi et al., 2020; Nadjib, 2020).

In addition to the mentioned medications, medicinal plants due to their natural origin have less complication than chemicals and therefore have received researchers’ attention during the coronavirus epidemic. Nowadays, medicinal plants are considered one of the most valuable sources of natural compounds for use in the production of antimicrobial and antioxidant drugs (Papari Moghadam Fard et al., 2021) as they have anticonvulsant, antipyretic, anti-inflammatory, and antimicrobial properties (Mani et al., 2020).

The treatment of viral infections using the available drugs has been accompanied by failure because of the drug resistance observed due to the mutagenesis of viruses, as a result of which, mankind needs to figure out new antiviral compounds. The best antiviral drug should properly function in specific stages of viral biosynthesis. In fact, they should function by introducing certain processes in the virus replication cycle to prevent the virus replication (Reichling et al., 2009).

In this regard, according to the results of previous studies, many plants such as colchicine* (Colchicum autumnal *L.*)*, olive (*Olea europaea *L.*) *leaf, black cumin (*Nigella sativa *L.*)*, lavender* (Lavandula angustifolia* L.), and ginger rhizome* (Zingiber officinale *Roscoe*)* with different composition ratios can have different effects on various types of viruses such as influenza, herpes, SARS, middle East respiratory syndrome (MERS), and COVID-19 (Koshak et al., 2020; Montealegre-Gómez et al., 2021; Reyes and Hu, 2021; Ballabeni et al., 2004; Bordoni et al., 2019; Tavakoli and Aryaeian, 2016; Omar, 2010; Hashem-Dabaghian et al., 2022; Schlesinger et al., 2020). 

In the light of interpreting these results, it can be stated that colchicine is an antiviral and anti-inflammatory drug recognized in the medical world for a long time. This medicinal product is obtained from *Colchicum autumnal *L. used to treat familial Mediterranean fever, gout, amyloidosis, and Behcet's syndrome (Papadopoulos et al., 2020; Scarsi et al., 2020). Moreover, it can also control inflammatory reactions through different pathways such as the inhibition of neutrophil chemotaxis, super-oxidants, and TNF (Schlesinger et al., 2020).

In addition, *Zingiber officinale *L. with its active chemical compounds such as 6-gingerol, 6-shogaol, and gingerol lessens inflammatory factors like cytokines and chemokines, reduces oxidative enzyme, and thus decreases the incidence and complications of inflammatory diseases by affecting inflammatory, antioxidant, and anti-serotonin mechanisms (Liao et al., 2012).

In clinical studies, olive (*Olea europaea *L.) leaf has had a very good effect on the treatment of hemorrhoids and vein thrombosis in the leg (Lockyer et al., 2015). Moreover, it has good inhibitory effects on NAPDH-oxidase, superoxide, IL-8, and NF-KB (Lockyer et al., 2015). 

In addition to the mentioned medicinal plants, *Lavandula angustifolia* L. has been used as a sedative, anti-depressant, anti-convulsant, and anti-inflammatory plant since ancient times. This plant has beneficial roles in modulating the immune system and inhibiting pro-inflammatory factors (such as Th17, IL-81, IL-1B, and TNF) and inflammatory factors like Th1 (interferon gamma and IL-2) and Th2 (IL-4, IL-5, IL-6 and IL-13), and IL-10 and inhibiting cytokines and the accumulation of inflammatory cells (Hashem-Dabaghian et al., 2022). 

Furthermore, *Nigella sativa *L. has been used to treat rheumatoid arthritis, asthma, inflammatory diseases, gastrointestinal tract, and diabetes. It is also believed to have the ability to block ACE and to be effective in the treatment of COVID-19 (Khazdair et al., 2021; Abbasnezhad et al., 2019).

Therefore, based on the available evidence and previous pertinent literature, the use of some medicinal plants such as colchicum bulb*, *olive leaf*, *black cumin seeds*, *lavender flower,* and *ginger rhizome can be hypothesized to be effective in reducing the symptoms of patients with COVID-19. Therefore, the present study aimed at evaluating the adjuvant therapy of this edible herbal combination on the recovery and outcome of patients with severe and critical COVID-19.

## Materials and Methods

### Study design

The current study was a randomized double-blind, placebo-controlled clinical trial. The study population included all patients with severe and critical COVID-19 admitted to the department of infectious diseases and the intensive care unit (ICU) of Al-Zahra and Amin hospitals in Isfahan from September 2021 to April 2022.

### Patient enrollment

In this study, 150 patients (75 in each group) were enrolled based on the formula of the sample size comparing the two groups, at the confidence level of 99%, the test power of 90%, and considering the results of previous studies (Sandhu et al., 2020) reporting the percentage of mortality in the control and colchicine groups equal to 47.1% and 80.8%, respectively. 

The inclusion criteria consisted of the definite infection with COVID-19 determined according to the diagnostic criteria, the acute (severe) or critical phase of this disease, not receiving COVID-19 vaccine, the age range of 40-85 years, and the weight of over 50 kg. It is worth noting that patients in the severe stage with the progression of respiratory symptoms, tachypnea (respiratory rate (RR) ≥30 bpm), shortness of breath (Oxygen saturation percentage (SpO2) < 90% and partial pressure of oxygen (Pao2) / Fraction of inspired oxygen (Fio2) ≤ 300 mmHg), increased A-a gradient, and increasing lung involvement of 50% specified in CT scan were included in the study (Hashem-Dabaghian et al., 2022). Furthermore, patients in the critical phase with at least one of the symptoms of severe respiratory failure despite non-invasive oxygen therapy treatments, SpO2 ≤ 88%, signs of shock, in need of mechanical ventilation, or suffering from multiple organ failure were included in the study (Hashem-Dabaghian et al., 2022).

However, the patients were not included in the study in case of pregnancy, breastfeeding, presence of active ulcer in the gastrointestinal tract, use of digoxin, admission to the ward before the start of the study, and history of allergy to medicinal plants. 

In addition, the patients were excluded from the study and substituted in case of the discontinuation of taking the medicinal plant combination for any reason, or the patient refusal to continue the cooperation in this study. 

### Medicinal plant preparation

The maceration method was used in this study to extract medicinal plants including colchicum bulb, olive leaf, black cumin seeds, lavender flower, and ginger rhizome (with equal weight). After being grounded to mean particle size 125 micron, they were mixed with three times of their total weight in rapeseed oil and mixed at a temperature of 35-40 degree of Celsius under oxygen-free conditions with nitrogen gas flow for 10 days. Then, the oil extract was separated from the mixture by filter press under a pressure of 200 psi. In order to further concentrate the filtrate was again mixed with plant powder in the same proportion. Under the same conditions of the first stage, the process was repeated, and the extraction was done again after 10 days. 

The oil extract was mixed and homogenized at a temperature of 60°C for 8 hr with an equal weight of Astragalus honey with a sucrose test below 2%. Medicinal plant mixtures were analyzed by HPLC method for colchicine content (each capsule contains 0.04 mg colchicine). Finally, the herbal medicine was prepared in one-gram capsules such that each capsule contained 500 mg of honey and 500 mg of the oil extract of herbal ingredients (colchicum bulb, olive leaf, black cumin seeds, lavender flower, and ginger rhizome) with equal proportions.

### Intervention

**Figure 1:**
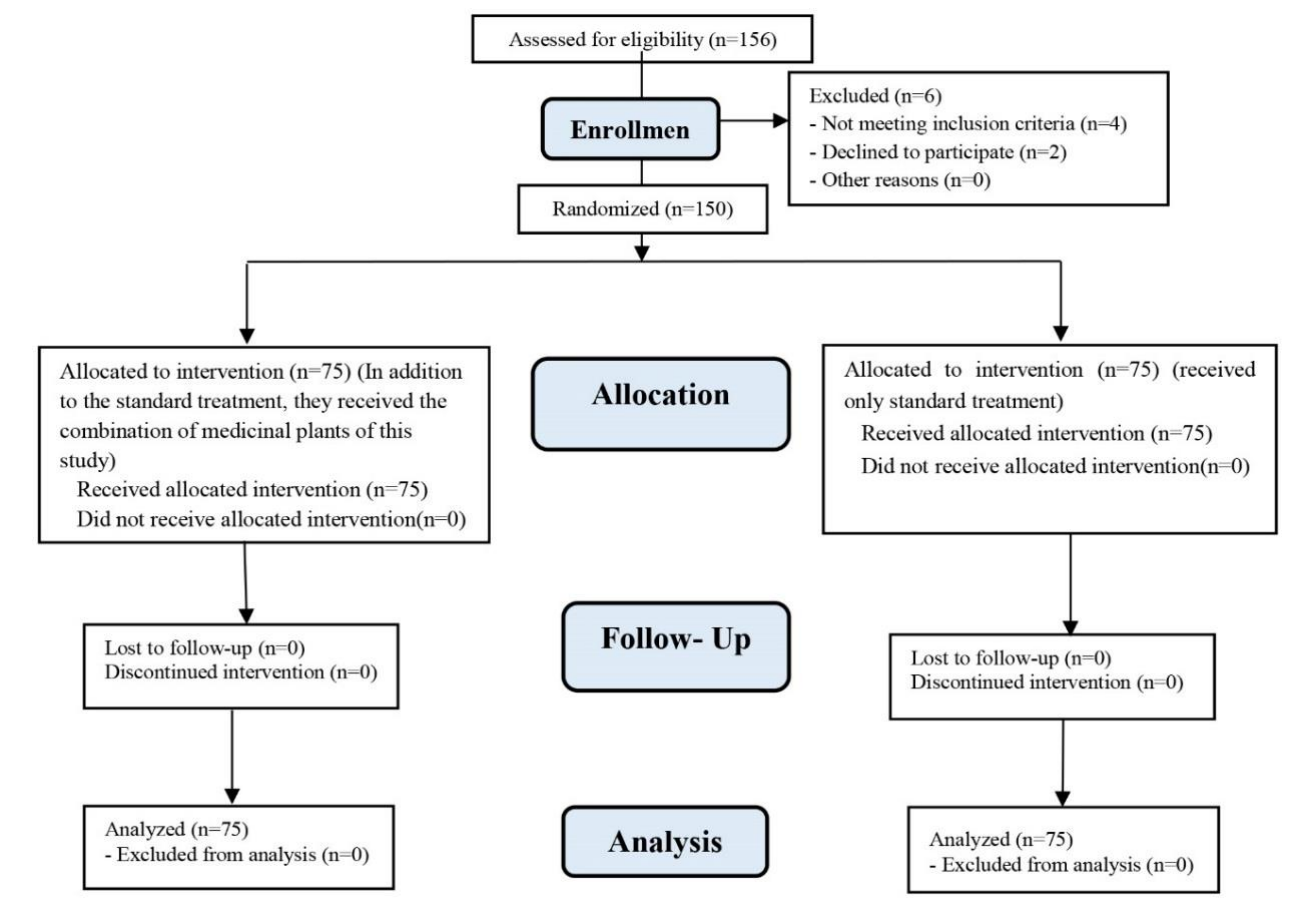
Consort flowchart of patients

After obtaining the approval from the ethics committee of Isfahan University of Medical Sciences (approval code: IR.MUI.MED.REC.1400.514), the clinical trial code (Code: IRCT20200825048515N49), and written consent from eligible patients or their companion, 150 patients were selected following simple random sampling method. Then, the patients were divided into two groups of 75 using the random allocation software. The flowchart of the study design is shown in Figure 1. 

At the beginning of the study, the patients’ demographic information including sex, age, body mass index (BMI), and co-morbidities including diabetes, blood pressure, cardiovascular diseases, chronic kidney diseases, chronic lung diseases, cancer, and immunodeficiency disorders were recorded. Moreover, their clinical data comprising initial symptoms and clinical manifestations such as fever, pulse rate (PR), RR, saturation of peripheral oxygen (SpO2), systolic blood pressure (SBP), and diastolic blood pressure (DBP) were recorded.

All patients received the standard care treatment of COVID-19 including pharmaceutical and supportive treatments such as oxygen therapy, remdesivir as an antiviral medication, corticosteroids, anticoagulants and tocilizumab (Actemra).

In addition to the standard care treatment, from the time of admission to the discharge of the patients, the first group as the intervention group received two capsules of herbal medicine orally every 8 hours. Moreover, if the patient was connected to a ventilator and oral administration of the medicine was not possible, two grams of the herbal medicine (equivalent to 2 ml) dissolved in 50 ml of warm water was given by gavage feeding. The second group, as the control group, received placebo medicine (1-gram capsules containing pure rapeseed oil and honey) with the same treatment protocol.

It is worthy of note that in order to comply with the conditions of blinding in the study, herbal medicine and placebo were prepared in advance by a medicinal plant specialist in the form of oral capsules in the same shape, size and color, coded with A and B labels, and provided to the researcher, who prescribed them to the groups without knowing the type of medicine. 

### Outcome measures

Patients’ hemodynamic, respiratory, and laboratory parameters comprising maximum and minimum blood pressure, SpO2, PR, RR, white blood cell (WBC), platelets (PLT), prothrombin time (PT), partial thromboplastin time (PTT), international normalized ratio (INR), lactate dehydrogenase (LDH), lymphocyte, erythrocyte sedimentation rate (ESR), C-reactive protein (CRP), D-dimer, aspartate aminotransferase (AST), alanine transaminase (ALT), alkaline phosphatase (ALP), blood urea nitrogen (BUN), creatinine (Cr), and ferritin were evaluated and recorded at the baseline and hospital discharge time.

Finally, the patient outcome (recovery, occurrence of any complications, oxygen requirement during discharge, or mortality) was checked and recorded for all patients at the end of the intervention. The criteria for recovery were the relieve of respiratory distress, the increase of blood oxygen level to above 93%, the absence of fever, and the reduction of inflammatory factors such as ESR and CRP. Moreover, the number of patients requiring hospitalization in the ICU, oxygen requirement duration, and hospital length of stay (LOS) were determined and recorded.

### Statistical analysis

Finally, the collected data were entered into IBM SPSS Statistics software (version 26, SPSS Inc., Chicago, IL, USA). Data are shown as means±standard deviation (SD), Number (percent), or median [IQR]. According to the results of the Shapiro-Wilk normality test indicating the abnormal distribution of clinical and laboratory parameters, the Mann-Whitney test was used to compare the mean of quantitative variables between the two groups at each of the study times, and in case of a significant difference in each of the variables at the baseline, the analysis of covariance (ANCOVA) was used to compare the variables at the time of discharge by adjusting the baseline variable. Moreover, the Wilcoxon test was used to compare the mean of quantitative variables at discharge as compared to the baseline in each of the two groups. Due to the normal distribution of variables such as age, BMI, LOS, the independent samples *t*-test was used to compare the mean of these variables between the two groups. The chi-squared test was used to compare the frequency distribution of qualitative variables between two groups. Moreover, survival analysis based on the Log Rank test was used to calculate the HR and the estimated survival time. In all analyses, the significance level of less than 0.05 was considered.

## Results

In the current study, there were 35 (46.7%) men and 40 (53.3%) women with the mean age of 67.85±14.35 years in the intervention group, while 33 (44%) men and 42 (56%) women with the mean age of 66.37±14.44 years were in the control group (p >0.05). Moreover, the percentage of hypertension was significantly higher in the intervention group as compared to the control group (60% vs. 40%; p=0.014). However, there was no significant difference between the two groups in terms of the other basic characteristics (p>0.05) (Table 1). 

Examining the vital signs as well as clinical and laboratory parameters of the patients in the two groups revealed that only fever and SpO2 level of the patients were significantly higher in the control group as compared to the intervention group at the beginning of the study (p <0.05). However, SpO2 in the intervention group (median [IQR]: 88.00 [85.00-90.00]) was significantly higher than SpO2 in the control group (median [IQR]: 86.00 [80.00-90.00]), while CRP in the intervention group (median [IQR]: 20.00 [8.00-35.00]) was significantly lower than CRP in the control group (median [IQR]: 28.00 [1.00-71.00]) at the time of hospital discharge (p <0.05). In addition, the results of comparing the changes of these parameters at the time of hospital discharge as compared to the beginning of the study indicated that PR, RR, D-dimer, CRP, AST, and Cr were significantly reduced whereas SpO2, PLT, and WBC were significantly increased in the intervention group. In the control group, D-dimer, CRP, and Cr were significantly decreased, while SpO2 was significantly increased (p <0.05). However, there were no significant differences in the other studied parameters between the two groups over time until hospital discharge (p >0.05) (Table 2). 

Furthermore, although there was no significant difference between the two study groups in terms of the incidence of dyspnea or oxygen requirement during hospitalization (p >0.05), the recovery time of dyspnea and the oxygen requirement duration were significantly lower in the intervention group with the means of 16.08±15.55 and 18.40±17.15 days, respectively as compared to the control group with the means of 34.66±21.01 and 35.37±22.44 days, respectively (p <0.001). Moreover, 6.7% of the patients in the intervention group and 29.3% of the control group were admitted to the ICU due to disease worsening (p <0.001). In this regard, all patients hospitalized in the ICU in the control group were connected to ventilators, while none of the patients hospitalized in the ICU in the intervention group required a ventilator. In addition, the oxygen requirement as non-invasive ventilation (NIV) or oxygen mask with a reservoir bag (OMR) in the control group with 11.1% and 23.8%, respectively was significantly more than the oxygen requirement in the intervention group with 2.7% and 9.3%, respectively at hospital discharge (p =0.005). The hospital LOS was significantly lower in the intervention group as compared to the control group (5.12±7.64 vs. 8.12±12.95 day; p <0.001). No hospital mortalities were reported in the intervention group, while 12 mortalities (16%) were recorded in the control group. Moreover, in the two-month follow-up after hospital discharge, 6 and 10 cases of mortalities were reported for the intervention group and control group, respectively (p-value<0.00) (Table 3). 

**Figure 2:**
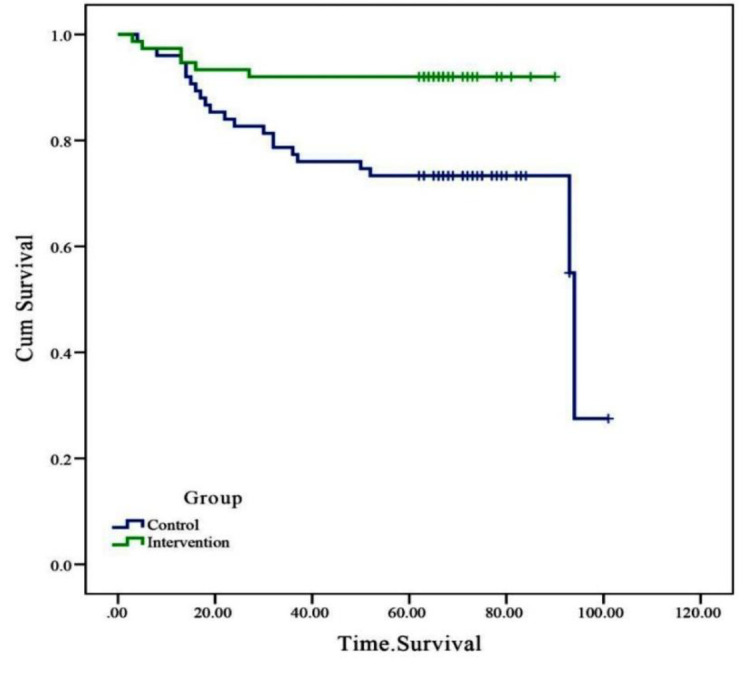
The survival curve of patients differed between the two study groups

Comparison of survival probability in the two groups based on the Log Rank test indicated that the estimated survival time in the intervention group with the mean of 83.83±2.43 days was significantly more than the control group with the mean of 76.78±4.13 days. In addition, the HR of mortality was much lower in in the intervention group as compared to the control group (HR [95% CI]: 0.29 [0.14-0.62]; p=0.003) (Table 4, Figure 2).

## Discussion

In this study, the combination of five medicinal plants with antiviral and anti-inflammatory effects was used to treat patients with severe or critical COVID-19. The results of this study revealed that although D-dimer, CRP, and Cr significantly decreased and SpO2 significantly increased in both groups, the patients receiving medicinal plant compounds containing *Colchicum autumnal *L., *Olea europaea *L.*,* *Nigella sativa *L., *Lavandula angustifolia* L., and *Zingiber officinale Roscoe* had higher SpO2 level and lower inflammation level (in terms of CRP parameter) than the control group at the time of hospital discharge. In other words, it can be stated that the present medicinal plant compound was effective in increasing the patients’ oxygen level and reducing their inflammation.

In line with the present study, various studies have been conducted regarding the anti-inflammatory and antiviral effects of each of the medicinal plants examined in this study. For example, Rathinavel et al. showed that 6-gingerol had a high tendency to bind to virus-infected cell proteins, including RNA polymerase, and prevented the viral replication and therefore could act as a hopeful drug of choice to treat COVID-19 (Rathinavel et al., 2020).

In our research, *Colchicum autumnal *L. was used as the most essential component of the herbal compound due to the presence of colchicine, which is one of the most important anti-inflammatory compounds in *Colchicum autumnal *L. In fact, the secretion of cytokines and all types of leukocytes are involved in mild and severe cases of the disease and fluid accumulation in the alveoli disrupt the function of epithelial cells and subsequently result in the occurrence of hypoxia (Ahmadi et al., 2021; Koshak and Koshak, 2020). Therefore, colchicine with its anti-inflammatory and antiviral effects can inhibit inflammatory reactions (Schlesinger et al., 2020) as neutrophils bind to epithelial cells in the first stage in many pathogen-induced inflammatory reactions. Microtubules play the most crucial role in binding neutrophils to epithelial cells. Therefore, they interfere with this process and inhibit inflammatory reactions by binding to colchicine (Scarsi et al., 2020; Schlesinger et al., 2020). Moreover, Colchicine reduces the TNFa factor of macrophages and also blocks the receptors on the endothelial surface layer. Consequently, the induction of NF-κB by TNF is significantly reduced when microtubules are destabilized by colchicine (Papadopoulos et al., 2020; Sandhu et al., 2020).

In addition, according to Hashem-Dabaghian et al.’s study, *Lavandula angustifolia* L. syrup has been an effective treatment for improving olfactory dysfunction (OD) (Hashem-Dabaghian et al., 2022). Actually, this plant has some compounds such as polyphenols and esters, which are well-known for their anti-inflammatory properties (Cavanagh and Wilkinson, 2002; Basch et al., 2004). Therefore, *Lavandula angustifolia* L. may be effective in OD due to its anti-inflammatory activities. 

Moreover, many studies have introduced *Nigella sativa *L. to have beneficial effects on the treatment or control of COVID-19 and other inflammatory and chronic diseases due to its anti-inflammatory, antioxidant, antimicrobial, neuro-protective, and reno-protective properties (Khazdair et al., 2021; Hajhashemi et al., 2004; Khazdair, 2015).

In addition, a mini review by Abdelgawad on the effect of *Olea europaea *L. leaves on the treatment of COVID-19 reported the presence of triterpenoids such as ursolic, oleanolic acids, and maslinic as well as phenolic-rich compounds such as apigenin-7-O-glucoside, hydroxytyrosol, luteolin-7-O-glucoside, oleuropein, and verbascoside in *Olea europaea *L. leaves. Recent *in vitro* and computational studies have reported the mentioned ingredients as anti-SARS-CoV-2 metabolites. Moreover, immunomodulatory, analgesic, antipyretic, antithrombotic, and anti-inflammatory activities have been described for the olive leaf extract in numerous *in vivo* studies. Controlling disseminated intravascular coagulation and the associated inflammatory cytokine storm are facilitated due to the stated activities (Abdelgawad et al., 2022). 

Therefore, reviewing the previous studies and the medicinal properties of the studied plants suggests that the patient can have a better outcome by controlling the virus and inhibiting the progress of inflammatory factors in the course of the COVID-19 disease. In this regard, the results of this study showed that a significant percentage of patients in the control group required hospitalization in the ICU due to the worsening of their disease such that all these patients were also connected to the ventilator, while the intervention patients hospitalized in the ICU did not need to be connected to the ventilator. Furthermore, the oxygen requirement as NIV or OMR at the time of hospital discharge was significantly higher in the control group as compared to the intervention group. In addition, the use of herbal compounds in this study resulted in a significant decrease in the recovery time of dyspnea and the oxygen requirement duration in the intervention group as compared to the control group. Moreover, no hospital mortality and six mortalities two months after discharge were reported for the intervention group, while twelve hospital mortalities and ten mortalities two months after discharge were recorded for the control group. Actually, according to the results of the survival analysis, the survival chance and time were far higher in the intervention group as compared to the control group.

Khazdair et al. stated that *Nigella sativa *L. can improve lung function in restrictive lung diseases due to its bronchodilatory effects as well as the anti-inflammatory and antiviral properties (Khazdair et al., 2021). Therefore, it is expected that patients receiving this plant require less oxygen therapy. 

It has also been reported that the administration of colchicine in the successful outpatient treatment of COVID-19 can greatly reduce the complications, the mortality rate, the need to be connected to a ventilator, and the necessity of biological treatments (Reyes and Hu, 2021). 

In fact, it can be stated that observing the medicinal plant contraindications and paying attention to their drug interaction effects can much better manifest the effectiveness of these medicinal plants with no complications. In this regard, *Zingiber officinale *Roscoe, *Nigella sativa *L*.*, *Lavandula angustifolia* L., and *Olea europaea *L. leaf are non-toxic herbal medicines (Idm’hand et al., 2020). If the extracts of mentioned medicinal plants are extracted with strong solvents such as ethanol, normal hexane, or methanol, they might cause poisoning. However, maceration in edible oils enters limited metabolites into the extract and do not cause strong drug interactions (Borges et al., 2020). Therefore, the present study used maceration, which can be considered one of the strong points of the study. Rapeseed oil was selected for extraction due to its high smoking point, high resistance to heat, and low oxidation (Rousseau, 2004). Among the most important chemical compounds used in this study were alkaloids, phenols, saponins, tannins, glucosides, essential oils, and terpenes. It should be noted that among these factors, oily compounds and terpenoids were lipophilic and more absorbed in oil than other compounds, and the effective ingredients for making this drug were extracted with minimal adverse changes. As oil extracts are unstable and change unfavorably over time, honey was used in this study to stabilize and thicken the extract and prevent its oxidation. This is another strength of this study. In addition, paying attention to patient’s conditions such as pregnancy, breastfeeding, gastrointestinal problems, use of digoxin, and a history of allergy to medicinal plants as exclusion criteria to prevent any complications caused by the use of these medicinal plants can be another strong point of this study. However, the small sample size and the non-evaluation of the effect of each of these drugs alone can be mentioned as the limitations of this study. Therefore, it is recommended to conduct future studies similar to the present study with a larger sample size examining different combinations of these medicinal plants.

According to the results of the present study, although the SpO2 level increased and the CRP level decreased significantly in both groups, the evaluation of the patients at the time of hospital discharge showed that the administration of the adjuvant therapy combination of five medicinal plants had a greater effect on increasing patients’ oxygen level and reducing their inflammation. In addition, prescribing the combination of medicinal plants in this study could significantly reduce the oxygen requirement, oxygen therapy need during hospital discharge, hospitalization in ICU, connection to the ventilator, and the hospital LOS. The survival chance and time were far higher in the intervention group as compared to the control group. In fact, it can be stated that the use of the combination of medicinal plants used in this study as a therapeutic supplement along with the standard care treatment can play a significant role in more successful treatment of COVID-19 by reducing the inflammation and increasing the oxygen level along with less complications.

**Table 1:** Patients’ basic characteristics in the two groups

**Characteristics**		**Intervention group (n=75)**	**Control group (n=75)**	**p-value**
Age; year		67.85±14.35	66.37±14.44	0.530
Sex	Male	35(46.7)	33(44.0)	0.743
	Female	40(53.3)	42(56.0)	
BMI; kg/m^2^		25.95 [23.87 - 27.97]	24.94 [23.57 - 27.54]	0.273
Underweight; BMI<18.5 kg/m^2^		0	1(1.6)	0.317
Healthy; 18.5≤BMI≤24.9 kg/m^2^		25(37.3)	31(50)	
Overweight; 25≤BMI≤29.9 kg/m^2^		32(47.8)	22(35.5)	
Obese; BMI≥ 30 kg/m^2^		10(14.9)	8(12.9)	
Comorbidities				
	DM	25(33.3)	28(37.3)	0.733
	HTN	45(60.0)	30(40.0)	0.014
	HLP	7(9.3)	6(8.0)	0.772
	CVA	5(6.7)	8(10.7)	0.384
	Asthma	3(4.0)	3(4.0)	0.999
	IHD	11(14.7)	12(16.0)	0.821
Medical Therapy				
	Anticoagulants	58(77.3)	53(70.7)	0.836
	Corticosteroids	63(60.0)	52(46.7)	0.102
	Remdesivir	23(30.7)	19(25.3)	0.467
	Actemra	3(4.0)	3(4.0)	0.999

Data presented as Mean±SD, Median [IQR] and Number (Percent). p-values calculated by Independent Sample T-test, Mann-Whitney U test, and Chi-square test.BMI: Body Mass Index; DM: Diabetes Mellitus; HTN: Hypertension; HLP: Hyperlipidemia; CVA: Cerebrovascular accident; IHD: Ischemic Heart Disease.

**Table 2:** Patients’ vital signs, clinical parameters, and laboratory parameters in the two groups

**Variables**		**Intervention group (n=75)**	**Control Group (n=75)**	**p-value** ^1^
**Vital signs and respiratory**				
T; °C	Baseline	36.50[36.10-37.00]	36.90[36.50-37.00]	0.011
	Discharge	36.50[36.40-37.00]	36.80[36.40-37.00]	0.100^†^
p-value^2^		0.537	0.287	
PR; bpm	Baseline	84.00[75.00-92.00]	84.00[79.00-89.00]	0.886
	Discharge	76.00[70.00-84.00]	82.00[76.00-88.00]	0.001
p-value^2^		<0.001	0.480	
RR; bpm	Baseline	21.00[20.00-22.00]	21.00[20.00-22.00]	0.531
	Discharge	20.00[20.00-21.00]	21.00[20.00-22.00]	0.058
p-value^2^		<0.001	0.875	
SBP; mmHg	Baseline	120.00[110.00-130.00]	120.00[110.00-130.00]	0.583
	Discharge	120.00[110.00-140.00]	120.00[110.00-130.00]	0.430
p-value^2^		0.198	0.354	
DBP; mmHg	Baseline	80.00[70.00-90.00]	75.00[70.00-90.00]	0.145
	Discharge	80.00[70.00-90.00]	75.00[70.00-80.00]	0.108
p-value^2^		0.849	0.815	
SpO2^*^; %	Baseline	83.00[80.00-85.00]	85.00[80.00-88.00]	0.026
	Discharge	88.00[85.00-90.00]	86.00[80.00-90.00]	<0.001^†^
p-value^2^		<0.001	<0.001	
Coagulation parameters				
PLT; ×10^9^/L	Baseline	195.00[145.50-253.00]	198.50[149.25-249.50]	0.733
	Discharge	224.00[163.50-301.00]	212.50[151.25-270.75]	0.281
p-value^2^		<0.001	0.129	
Fer; μg/L	Baseline	226.50[136.75-622.25]	339.00[149.00-912.00]	0.184
	Discharge	226.50[136.75-622.25]	343.00[149.00-912.00]	0.184
p-value^2^		0.999	0.317	
LDH; U/L	Baseline	565.00[425.50-826.00]	643.00[514.00-744.50]	0.245
	Discharge	536.00[424.50-746.50]	643.00[484.00-802.50]	0.202
p-value^2^		0.308	0.953	
D-Dimer; ng/ml	Baseline	618.00[370.25-1252.00]	753.50[396.50-1347.00]	0.185
	Discharge	524.00[383.00-998.75]	732.00[387.50-1316.25]	0.119
p-value^2^		0.018	0.034	
PT; s	Baseline	11.55[10.00-13.37]	12.10[10.85-14.05]	0.109
	Discharge	11.55[10.37-13.07]	12.10[11.00-14.20]	0.069
p-value^2^		0.999	0.836	
PTT; s	Baseline	28.00[26.15-30.00]	29.00[27.00-31.75]	0.134
	Discharge	28.00[26.37-31.00]	29.00[27.00-31.75]	0.237
p-value^2^		0.069	0.900	
INR	Baseline	1.13[1.01-1.25]	1.13[1.04-1.27]	0.789
	Discharge	1.11[1.00-1.25]	1.13[1.05-1.27]	0.404
p-value^2^		0.528	0.820	
Inflammatory parameters					
WBC; ×10^9^/L	Baseline	7.70[5.10-11.15]	8.90[6.10-11.47]	0.237
	Discharge	8.90[7.15-11.60]	9.30[6.55-12.17]	0.729
p-value^2^		0.001	0.351	
Lymph; %	Baseline	13.40[8.00-19.85]	12.20[8.00-18.62]	0.687
	Discharge	14.00[8.45-19.50]	10.65[6.27-19.70]	0.073
p-value^2^		0.736	0.171	
CRP; mg/L	Baseline	58.00[29.00-87.00]	65.00[22.00-88.00]	0.913
	Discharge	20.00[8.00-35.00]	28.00[1.00-71.00]	0.042
p-value^2^		<0.001	<0.001	
ESR; mm/hr	Baseline	34.00[15.20-66.50]	39.50[18.25-70.00]	0.790
	Discharge	32.00[13.50-62.75]	38.00[18.25-62.75]	0.610
p-value^2^		0.063	0.136	
Liver parameters					
AST; U/L	Baseline	30.50[25.00-43.25]	31.50[22.00-49.75]	0.909
	Discharge	28.50[21.75-38.00]	27.00[20.25-44.75]	0.624
p-value^2^		0.044	0.050	
ALT; U/L	Baseline	23.50[15.00-38.00]	26.00[16.00-37.00]	0.959
	Discharge	26.00[19.75-45.00]	26.50[17.00-46.50]	0.680
p-value^2^		0.180	0.410	
ALP; U/L	Baseline	159.00[134.00-194.00]	169.50[133.50-238.00]	0.281
	Discharge	163.00[132.50-197.50]	165.00[133.00-222.50]	0.481
p-value^2^		0.865	0.142	
Renal parameters					
BUN; mg/dL	Baseline	21.00[15.00-30.00]	22.00[13.00-37.00]	0.876
	Discharge	24.00[17.00-34.00]	25.00[16.00-34.00]	0.927
p-value^2^		0.185	0.081	
Cr; mg/dL	Baseline	1.00[0.90-1.30]	1.00[0.90-1.40]	0.806
	Discharge	1.00[0.80-1.10]	1.00[0.80-1.20]	0.976
p-value^2^		0.038	0.001	

Data are shown as median [Interquartile Range (IQR)]. *: Without the use of oxygen support. 1: The significance level of the Mann-Whitney test to compare the mean of variables between two groups in each of the studied times. †: The significance level of ANCOVA to compare the mean of the variables between the two groups by adjusting the baseline values as covariates. 2: The significance level of Wilcoxon Signed-Rank Test to compare the mean of the variables at the time of hospital discharge as compared to the baseline in each of the two groups. T: Temperature; PR: Pulse Rate; RR: Respiratory Rate; SBP: Systolic Blood Pressure; DBP: Diastolic Blood Pressure; SpO2: Oxygen Saturation; PLT: Platelets; Fer: Ferritin; LDH: Lactate Dehydrogenase; PT: Prothrombin Time; PTT: Partial Thromboplastin Time; INR: International Normalized Ratio; WBC: White Blood Cells; Lymph: Lymphocyte; CRP: C-Reactive Protein; AST: Aspartate Aminotransferase; ALT: Alanine Aminotransferase; ALP: Alkaline Phosphatase; BUN: Blood Urea Nitrogen; Cr: Creatinine.

**Table 3:** Evaluation of patients’ outcome in the two groups

**Outcome**	**Intervention group (n=75)**	**Control group (n=75)**	**p-value**
Dyspnea	38(50.7)	32(42.7)	0.398
Dyspnea time; day	16.08±15.55	34.66±21.01	<0.001
Oxygen requirement	37(49.3)	31(41.3)	0.471
Oxygen requirement duration; day	18.40±17.15	35.37±22.44	<0.001
Oxygen requirement status at discharge			
NIV(BiPAP)	2(2.7)	7(11.1)	
OMR	7(9.3)	15(23.8)	0.005
Nasal mask	64(85.3)	41(65.1)	
No Oxygen needed	2(2.7)	0	
ICU admission	5(6.7)	22(29.3)	<0.001
LOS; day	7.64±5.12	12.95±8.12	<0.001
Hospital Mortality	0	12(16.0)	<0.001
Cumulative mortality at 2 months follow-up	6(8.0)	22(29.3)	0.001

Data presented as Mean±SD, Median [IQR] and Number (Percent). p-values calculated by Independent Sample T-test, chi-square and fisher exact test. NIV: Non-Invasive Ventilation; BiPAP: Bilevel Positive Airway Pressure; OMR: Oxygen Mask with a Reservoir bag; ICU: Intensive Care Unit; LOS: Hospital Length of Stay

**Table 4:** Comparison of survival analysis in the two groups

Survival Analysis	Intervention group (n=75)	Control group (n=75)	Overall (n=150)
Estimated survival time; day	83.83±2.43	76.78±4.13	83.24±2.95
95% CI	79.06-88.59	68.68-84.88	77.46-89.01
HR (95% CI)	0.29(0.14-0.62)	Reference	
Chi Squared^ a^ = 8.542; df =1; p value = 0.003			

HR: Hazard ratio; CI: Confidence Interval. a: According to Log Rank (Mantel-Cox) test
